# Development of an Exhaled Breath Monitoring System with Semiconductive Gas Sensors, a Gas Condenser Unit, and Gas Chromatograph Columns

**DOI:** 10.3390/s16111891

**Published:** 2016-11-10

**Authors:** Toshio Itoh, Toshio Miwa, Akihiro Tsuruta, Takafumi Akamatsu, Noriya Izu, Woosuck Shin, Jangchul Park, Toyoaki Hida, Takeshi Eda, Yasuhiro Setoguchi

**Affiliations:** 1 National Institute of Advanced Industrial Science and Technology (AIST), Shimo-Shidami, Moriyama-ku, Nagoya 463-8560, Japan; t-miwa@glc.shimadzu.co.jp (T.M.); a.tsuruta@aist.go.jp (A.T.); t-akamatsu@aist.go.jp (T.A.); n-izu@aist.go.jp (N.I.); w.shin@aist.go.jp (W.S.); 2 Aichi Cancer Center, 1-1 Kanokoden, Chikusa-ku, Nagoya 464-8681, Japan; samasama75@gmail.com (J.P.); 107974@aichi-cc.jp (T.H.); 3 Figaro Engineering Inc., 1-5-11 Sembanishi, Minoh, Osaka 562-8505, Japan; eda@figaro.co.jp (T.E); setoguchi@figaro.co.jp (Y.S.)

**Keywords:** lung cancer, volatile organic compounds, exhaled breath analysis, gas condenser-equipped gas chromatography-mass spectrometry, condenser, GC, sensor-type prototype system

## Abstract

Various volatile organic compounds (VOCs) in breath exhaled by patients with lung cancer, healthy controls, and patients with lung cancer who underwent surgery for resection of cancer were analyzed by gas condenser-equipped gas chromatography-mass spectrometry (GC/MS) for development of an exhaled breath monitoring prototype system involving metal oxide gas sensors, a gas condenser, and gas chromatography columns. The gas condenser-GC/MS analysis identified concentrations of 56 VOCs in the breath exhaled by the test population of 136 volunteers (107 patients with lung cancer and 29 controls), and selected four target VOCs, nonanal, acetoin, acetic acid, and propanoic acid, for use with the condenser, GC, and sensor-type prototype system. The prototype system analyzed exhaled breath samples from 101 volunteers (74 patients with lung cancer and 27 controls). The prototype system exhibited a level of performance similar to that of the gas condenser-GC/MS system for breath analysis.

## 1. Introduction

Air exhaled by humans contains many volatile organic compounds (VOCs) that are associated with metabolism [1,2], mouth odor [3], and diseases. Some VOCs in the exhaled breath may be biomarkers for diseases, such as cancer [4]. Early detection and prompt treatment of lung cancer is critical for achieving good outcomes. Although annual chest X-ray radiography (CXR) is frequently used for screening of lung cancer and tuberculosis, the use of CXR for early detection of lung cancer has not yet been supported by published evidence [5]. To improve screening accuracy, low-dose computed tomographic (LDCT) scanning has been investigated for lung cancer detection [5,6,7,8,9]. However, this procedure involves risks associated with patient exposure to radiation [6,9].

Monitoring of exhaled breath is one of the most noninvasive screening techniques for early diagnosis; however, this method is limited by insufficient accuracy, as many VOCs are present in the exhaled breath at very low concentrations (ppb level). Although gas chromatography-mass spectrometry (GC/MS) is one of the best methods for detecting low-concentration VOCs, this method is expensive, and the instrumentation is not portable; inexpensive detectors, such as portable VOC detectors, are preferable for periodic medical inspection and health screening. Many researchers have developed systems to analyze exhaled breaths, including multisensor signals from sensor arrays that utilize statistical analysis [10,11,12,13,14,15,16,17,18,19,20,21,22,23,24,25,26], ion mobility spectrometry [27], and discrimination by canines [28,29]. Accordingly, we have developed a prototype system for monitoring exhaled breath. The prototype system has SnO_2_-based semiconductor gas sensors as detectors and is equipped with a simple gas condenser unit and simple GC columns. In this system, the exhaled breath is aspirated from a gasbag for condensation of VOCs in the gas condenser unit, and the gas condenser unit is then heated for desorption of VOCs. Subsequently, the VOCs are applied to the GC columns for separation and are then detected automatically by the gas sensors. Therefore, this system has the following advantages: simple operation, no requirement for specialized knowledge and experience, and potential for adjustment of the monitored VOCs by changing the GC column. SnO_2_ is one of the best materials for VOC sensors because of its high sensitivity. For example, Pt-loaded SnO_2_ nanowires show effective selectivity to 1 ppm toluene as a lung cancer-related VOC [30], and open pores of SnO_2_ surfaces induce strong responses to toluene [31]. In the prototype system, the GC columns function in selectivity; thus, the semiconductive gas sensors function as detectors. Therefore, the semiconductive gas sensors should possess the ability to detect various groups of VOCs. Pristine SnO_2_ shows lower responses to aliphatic, halogenated, and aromatic hydrocarbons than to other groups of VOCs, such as oxy-hydrocarbons. Moreover, the addition of Pt, Pd, and Au to SnO_2_ thick films is effective for improving the sensitivity of these systems to aliphatic, halogenated, and aromatic hydrocarbons, respectively [32,33,34,35]. Additionally, Pt, Pd, and Au/SnO_2_, with optimized film thickness and pretreatment conditions, i.e., annealing and aging, show high sensitivity to nonanal gas at the several tens ppb level without any gas condensation system [36]. Thus, Pt, Pd, and Au/SnO_2_ may be suitable detectors for GC with gas condenser-type prototype systems.

Biomarker VOCs of lung cancer from the exhaled breath [15,21,28,37,38,39,40,41,42,43,44,45] and headspace of lung cancer cell lines [15,19,24,41,42,46] have been extensively studied. These studies have reported various biomarker VOCs in lung cancer, including alkanes [38,44,45,47,48], aromatic hydrocarbons [19,28,38,39,44,45,47,48], and aldehydes [19,28,37,38,46]. The use of VOCs as biomarkers has also been summarized in several papers [47,49,50]. However, various compounds have been reported as optimal biomarkers, suggesting disagreement among many previous studies. For these reasons, VOC patterns, i.e., not single VOCs, but combinations of several VOCs, should be used for exhaled breath analysis for the diagnosis of different diseases [50]. Additionally, the prototype system, which adopted semiconductive gas sensors, a gas condenser, and GC columns, was not expected to be able to cover all biomarker VOCs reported in lung cancer to date. Moreover, differences in VOC profiles and concentrations may arise from differences in analytical conditions between studies, including differences in sample collection, preconcentration of breath VOCs, and measurement techniques [15,47], such as solid-phase micro-extraction with GC/MS (SPME-GC/MS) [11,12,14,19,21,24,28,37,39,41,42,43,44,45] and proton transfer reaction mass spectroscopy (PTR-MS) techniques [39]. Therefore, the use of a gas condenser-equipped GC/MS may provide optimal results in the analysis of lung cancer-related VOCs for development of GCs with gas condenser-type prototype systems.

In the present study, we developed a GC with a gas condenser-type prototype system for VOC detection in breath exhaled by patients with lung cancer and evaluated the performance of the prototype system by comparison with the gas condenser-GC/MS. 

## 2. Experimental Section

### 2.1. Selection of Gas Bags

We collected ambient air from a consultation room at Aichi Cancer Center using an Analytic Barrier bag (Omi Odor-air Service Corp., Omi, Japan) and a cleaned Tedlar bag (GL Science, Tokyo, Japan). The Tedlar bag has been used for breath collection in previous reports [10,22,28,39,41,42,43]. The Analytic Barrier bag possessed low permeation and adsorption properties, similar to the Tedlar bag, as described by the manufacturer. We then analyzed the two samples by GC/MS.

### 2.2. Collection of Exhaled Breaths and Gas Condenser-GC/MS Analysis

After approval by the local ethics committee of Aichi Cancer Center and AIST and obtaining written informed consent, 107 patients with lung cancer and 29 healthy controls were enrolled in this study. Participating patients with lung cancer received a diagnosis of lung cancer between 1 March 2012 and 30 November 2012 at Aichi Cancer Center. Exhaled breaths were collected from 44 patients at 6–8 weeks after surgical tumor resection. These 44 patients who underwent resection received a diagnosis of early-stage lung cancer before surgery and consented to the collection of exhaled breaths after surgery. All volunteers did not eat or smoke for several hours before collection of samples. Moreover, before breath collection, all volunteers stayed in the consultation room for at least 10 min and in the waiting room at the Aichi Cancer Center for at least 30 min prior to entering the consultation room. All volunteers blew their alveolar breath into Teflon stopcocks of 1 L Analytic Barrier bags directly after they exhaled their respiratory tract air in the consultation room. The stage and histology of the lung cancer samples are summarized in Table 1.

The gas condenser-GC/MS data were acquired using a GCMS-QP2010 instrument (Shimadzu, Kyoto, Japan) equipped with a TD-2 gas-condensing unit (Shimadzu, Kyoto, Japan). The TD-2 has a gas aspiration unit and a cold trap for condensation of low-concentrate VOCs. The GC/MS system used helium gas of 99.9995% purity (Taiyo Nippon Sanso, Tokyo, Japan) as the carrier gas. Tygon tubing (Saint-Gobain, Paris, France) was used to connect the gasbag and the TD-2. Before the GC/MS analysis, the TD-2 unit aspirated the exhaled breath for 0.5 min at 60 mL/min for line purging, and the exhaled breath was then continuously aspirated for condensation of VOCs in a cold trap at −20 °C for 4 min. After condensation, the cold trap was heated quickly, and desorbed VOCs were applied to the GC/MS system. The GC/MS analysis was then started automatically. The GC column was a DB-1 series 123-1063 (Agilent Technologies, Santa Clara, CA, USA), whose interior diameter, film thickness, and length were 0.32 mm, 1 μm, and 60 m, respectively. The GC column was heated according to the following heating program: initial temperature of 35 °C for 5 min; increased at 10 °C/min to 175 °C; increased at 30 °C/min to 250 °C; and finally maintained at 250 °C for 3.5 min. The mass-to-charge ratio (*m*/*z*) was monitored between the values of 22 and 250. VOCs were identified using the mass database provided by Shimadzu. When the mass pattern of a peak was identified as several isomers, the VOC of the peak was expressed as a composition formula (e.g., C_8_H_17_OH). For quantitative comparisons, the peak areas of the GC/MS signals were normalized according to the peak of the toluene calibration gas (5 ppm), which served as an external standard because few calibration gases for all VOCs have been prepared; therefore, the concentration of all VOCs was provided as the toluene-equivalent concentration. Before the breath analyses, the 5 ppm toluene calibration gas was analyzed, and the peak area of 5 ppm toluene was determined. The peak areas of all peaks from the exhaled breath were then transformed to toluene-equivalent concentrations. In the calibration analysis, the TD-2 unit was used to aspirate the toluene calibration gas at 60 mL/min from the toluene gas flow in the Teflon tube. The signal-to-noise (S/N) ratio of the minimum limit of detection was set at 1.5, which corresponded to a peak toluene equivalent concentration of approximately 1 ppb, as recommended by manufacturer for the point of reliability. 

### 2.3. Statistical Analysis

**Figure 1:**
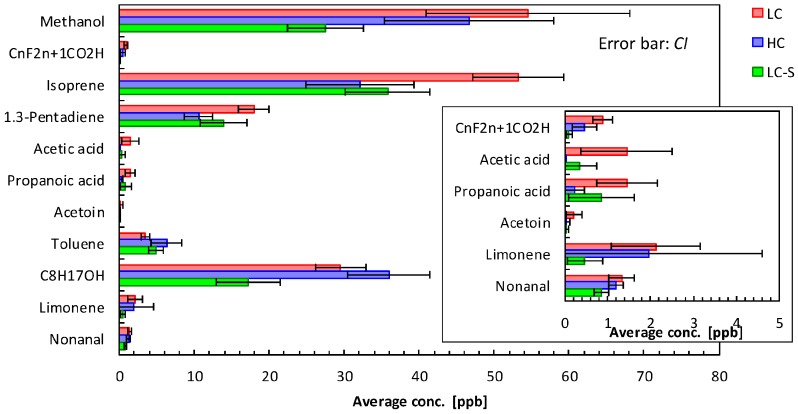
Average concentrations and confidence intervals (CIs) of 11 VOCs in exhaled air from LC, HC, and LC-S groups. For these 11 VOCs, the *p* value was less than 0.05 for differences between LC and HC groups and/or between LC and LC-S groups.

In the gas condenser-GC/MS analysis, 56 VOCs were detected and identified from 180 exhaled air samples. We carried out *t*-tests between LC and HC or LC and LC-S. Student’s or Welch’s *t*-tests were performed when the significance level of the population variance was over or below 0.05, respectively. In this test, we selected several VOCs that were significantly different in the exhaled air between LC and HC groups and/or LC and LC-S groups. A probability value (*p* value) of 0.05 was defined as significant. The selected VOCs by *t*-tests are presented as the average concentration with error bars of confidence intervals (CIs) in Figure 1.

To reduce the breadth of coverage from selected VOCs for the prototype system, we also carried out correlation coefficient analysis as an indicator of the intensity of the linear relationship between two random variables. The correlation coefficient, *r*, between the two candidate VOCs was defined as:

(1) $$r=\frac{S_{ab}}{\sqrt{S_{aa}\times S_{bb}}}$$

where

(2) $$S_{ab}=\sum_{i}\left(a_{i}-\bar{a}\right)\left(b_{i}-\bar{b}\right)$$

(3) $$S_{aa}=\sum_{i}{\left(a_{i}-\bar{a}\right)}^{2}$$

(4) $$S_{bb}=\sum_{i}{\left(b_{i}-\bar{b}\right)}^{2}$$

where $\bar{a}$ and $\bar{b}$ are the average concentrations of two VOCs, and *a_i_* and *b_i_* are the concentrations of two VOCs in the exhaled breath. The range of the correlation coefficient was −1 ≤ *r* ≤ 1. When the absolute value of the correlation coefficient was close to 1, the two random variables were in a strong linear relationship. In this study, combinations of VOCs with *r* values of greater than 0.6 from LC were selected to allow for selection of target VOCs for the prototype system.

### 2.4. Preparation of Sensor Elements

**Figure 2:**
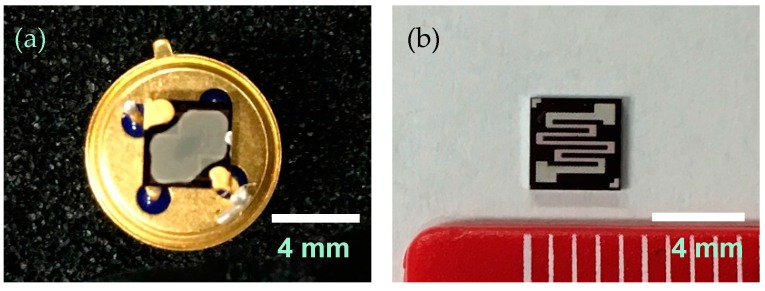
Pt, Pd, and Au/SnO_2_ sensor element: (**a**) front (Pt, Pd, and Au/SnO_2_ thick film with platinum comb-type electrode); and (**b**) back (platinum heater). The substrate size was 4 × 4 mm^2^.

The Pt-, Pd-, and Au-loaded SnO_2_ sensor elements were prepared as reported previously [36]. Figure 2 shows the front and back of the Pt, Pd, and Au/SnO_2_ sensor elements. A platinum heater was patterned on the back of a 4 × 4 mm^2^ surface-oxidized silicon substrate and a platinum comb-type electrode 7.5 mm^2^ in area with a 10 μm gap, and 10-μm line width was formed on the front of the substrate. Pt (particle size: 2 nm), Pd (4 nm), and Au (3 nm) colloid suspensions (Tanaka Kikinzoku Kogyo K.K., Tokyo, Japan) were added to the SnO_2_ powder (particle size: <100 nm; Aldrich, St. Louis, MO, USA) at 1 wt % each relative to SnO_2_. The mixtures were stirred, dried, and subsequently heated at 350 °C for 2 h. The resulting powder was combined with an ethylcellulose-type organic dispersant to obtain a paste. The powder/vehicle ratio of the paste was 1/16. The paste was subsequently applied to the electrode of the substrate using a FAD-320s dispenser (Musashi Engineering, Tokyo, Japan). The substrate was then dried at 80 °C for 2 h and annealed at 500 °C for 2 h in room air to obtain the Pt, Pd, and Au/SnO_2_ thick film. Using this preparation method for the Pt, Pd, and Au/SnO_2_ thick films, the film thicknesses were approximately 2.9 μm, as described previously [36]. The resulting substrates were mounted on dedicated stems. Next, the Pt, Pd, and Au/SnO_2_ thick films were held at 300 °C for 3 days in room air as aging to improve the sensitivity using a backside platinum heater before installing the substrates in the prototype system.

### 2.5. Prototype System

**Figure 3:**
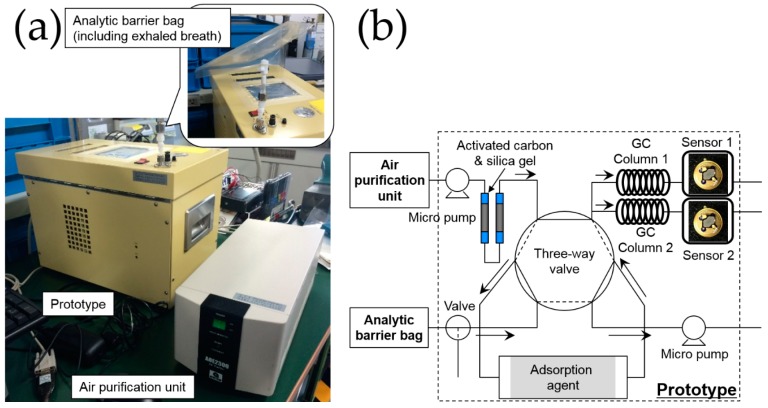
(**a**) Prototype system; and (**b**) flow stream of the prototype system.

Figure 3 shows the prototype system and its flow stream. The prototype system used a double column with detectors, similar to a GC instrument, for detecting four types of VOCs, e.g., nonanal, acetoin, and short-chain fatty acids. The two Pt, Pd, and Au/SnO_2_ sensor elements were installed into the prototype as detectors. For separation of four types of VOCs and to be sure the retention times of the four types of VOCs were within the total measurement time (10 min), GC column 1 was modified to have an inner diameter of 2 mm and a length of 60 cm, filled with styrene monomer-analyzing type column packing (Bentone 34 + SP-1200; GL-Science, Tokyo, Japan). GC column 2 was modified to have an inner diameter of 2 mm and a length of 50 cm, filled with short-chain fatty acid-analyzing type column packing (Unisole F-200; GL-Science, Tokyo, Japan). Tenax TA was used as the adsorbent agent (GL-Science, Tokyo, Japan); based on the breakthrough volume of Tenax TA for the four types of VOCs, 40 g of Tenax TA was added to a glass tube with an inner diameter of 3 mm and a length of 94 cm. The prototype system used room air as a carrier gas and had an AOE 2300 air purification unit (GL Science, Tokyo, Japan) and two activated carbon/silica gel purification units. In the standby state, purified room air was flowed into GC columns and sensor chambers at 77 mL/min; two Pt, Pd, and Au/SnO_2_ sensor elements were heated to 250 °C, and two GC columns were kept at 74 °C. In the VOC-condensing state, Tygon tubing was used to connect the Teflon stopcock of the Analytic Barrier bag and the prototype, and 200 mL of exhaled breath was aspirated for 120 s and condensed by Tenax TA at room temperature. After condensing, the Tenax TA was heated to 180 °C for 60 s for desorption. In the VOC-analysis state, a three-way valve was used to change the flow stream such that the purified room air flowed into the Tenax TA. Diffused VOC molecules from Tenax TA were flowed into two columns kept at 74 °C and detected by two sensor elements. The original GC counts were evaluated from the sensor signals of the Pt, Pd, and Au/SnO_2_, corresponding to the square of the change in output voltage, which depended on the resistance (Figaro Engineering Inc., Minoh, Japan). For the semiconductive gas sensors, the original count value was empirically proportional to the concentration of VOCs. For verification of the appropriate filling amount of column packing and adsorbent agent for breath analysis, we analyzed the prototype system using 2 mL of 1, 5, 10, and 30 ppm VOCs (for the four target VOCs), which were prepared from vaporization of the liquid sources of four VOCs in gasbags, for which the condensed amounts were almost the same as that of 200 mL (exhaled breath analysis, see Section 2.6) of 10, 50, 100, and 300 ppb VOCs, respectively. 

### 2.6. Collection of Exhaled Breaths and Prototype Analysis

After obtaining approval by the local ethics committee of Aichi Cancer Center and AIST and written informed consent from the participants, 74 patients with lung cancer and 27 healthy controls were enrolled in this study. Participating patients with lung cancer had received a diagnosis of lung cancer between 1 July 2015 and 20 January 2016 at Aichi Cancer Center. Breath samples from all volunteers were collected as described in Section 2.2. Before the first exhaled breath analysis, pure air was analyzed several times as dummy analyses for cleaning the flow stream of the prototype until the base signal was restored. As described in Section 2.5, 200 mL of exhaled breath was aspirated for condensation of VOCs in Tenax TA as the adsorbent agent. The Tenax TA was then heated, desorbed VOCs were applied to the two GC columns, and prototype analysis was started automatically. For every interval of the exhaled breath analyses, pure dry air was re-analyzed several times. 

### 2.7. Evaluation of the Performance of the Prototype

For evaluation of the performance of the prototype, we defined the cutoff VOC concentration value, *C*_cutoff_, which was the maximum concentration from HC. The *C*_cutoff_ of all target VOCs of the prototype was evaluated from the results of the gas condenser-GC/MS and the prototype. We corrected number of samples that included concentrations over the *C*_cutoff_ concentration and evaluated the performance of the prototype system by comparison with the results of gas condenser-GC/MS.

## 3. Results and Discussion

### 3.1. Selection of Gas Bags and Contamination of Room Air

**Figure 4:**
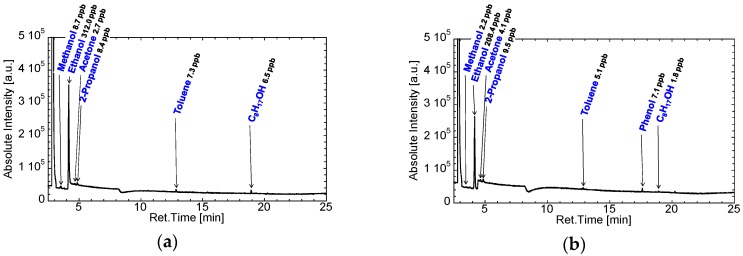
GC/MS spectra of the air of the consultation room in Aichi Cancer Center obtained using: an Analytic Barrier bag (**a**); and cleaned Tedlar bag (**b**).

Figure 4 shows the GC/MS spectra of the room air of the consultation room in Aichi Cancer Center collected using Analytic Barrier bags and the cleaned Tedlar bags. Tedlar bags should be cleaned by displacing pure nitrogen or argon and heating [28,39,42,43]; therefore, we purchased precleaned Tedlar bags. Phenol was detected from the Tedlar bag, and the other six VOCs were detected from both types of bags. Therefore, phenol should be considered a contaminant in the Tedlar bag. The concentration of phenol was similar to the concentration of the other VOCs, although the concentration of phenol was reduced by the cleaning treatment. We therefore selected the Analytic Barrier bags for the collection of exhaled air. The other six VOCs were expected to be from the room air and/or gasbags. The concentrations of these VOCs were excluded from the results of breath analysis in this study.

### 3.2. Gas Condenser-GC/MS Analysis of Exhaled Breaths for Development of the GC-Gas Sensor-Gas Condenser-Type Prototype

**Figure 5:**
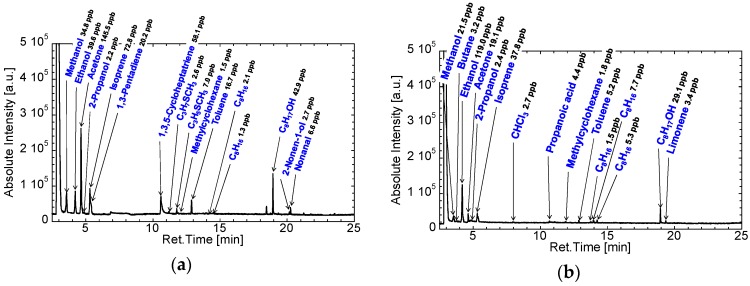

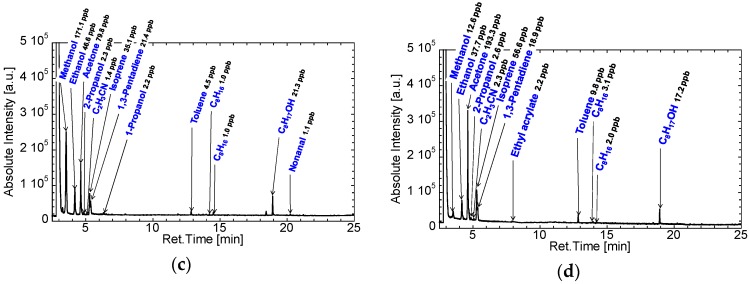
GC/MS spectra of exhaled breaths from: (**a**) a patient with lung cancer (IA, Ad); (**b**) another patient with lung cancer (IIIA, Ad); (**c**) a healthy control; and (**d**) the patient from (**b**) after undergoing surgery.

Figure 5a,b shows the GC/MS spectra of exhaled breaths from patients with lung cancer. The histology of lung cancer samples indicated that these samples were adenocarcinomas. The stages of lung cancer were IA and IIIA; the sample shown in Figure 5b (stage IIIA) was from a patient with a more advanced stage of cancer than that shown in Figure 5a (stage IA). However, the VOCs detected from the patient in Figure 5a were not necessarily detected from the patient in Figure 5b. Fuchs et al. reported that nonanal is a biomarker of lung cancer [37]. The concentration of nonanal in the breath sample from the patient in Figure 5a was 6.6 ppb, which was higher than the concentrations in exhaled breath samples from healthy controls (Figure 5c). In contrast, nonanal was not detected in the exhaled breath from the patient with lung cancer presented in Figure 5b. The exhaled breath of the patient with lung cancer presented in Figure 5b included propanoic acid, and the exhaled breath of the same patient after undergoing surgery did not include propanoic acid (Figure 5d). Moreover, propanoic acid was also not found in the exhaled breath of the patient with lung cancer presented in Figure 5a.

In the gas condenser-GC/MS analysis, 56 VOCs were detected and identified from 136 exhaled breath samples, as presented in Table 2. Their average concentrations and standard deviations are also included in Table 2. These results may also imply that a particular VOC is actually not a suitable biomarker for lung cancer. In this study, we also found that the exhaled breath from patients with lung cancer did not always include the same VOCs, although some of the exhaled breath samples contained some of these VOCs, including nonanal and propanoic acid. Moreover, some exhaled breath samples from patients with lung cancer included certain VOCs at higher concentrations than those observed in samples from healthy controls and patients with lung cancer who underwent surgery. 

Using Student’s and Welch’s *t*-tests, we evaluated 11 VOCs as candidate lung cancer-related VOCs by analysis of GC with the gas condenser unit. Figure 1 shows the average concentrations and confidence intervals of 11 VOCs in exhaled air from LC, HC, and LC-S. The error bars indicate the 95% confidence intervals; the error bars in the LC group did not overlap those of the HC and/or LC-S groups; therefore, the *p* value was less than 0.05 for the differences between the LC and HC groups and/or between the LC and LC-S groups for the 11 VOCs. 

In previous studies, many researchers have used SPME-GC/MS to study biomarkers of lung cancer [14,28,37,39,41,42,43,44,45]. Here, we evaluated the results from the gas condenser-GC/MS and compared these results with those from SPME-GC/MS. Peng et al. reported that 42 VOCs could be used as biomarkers of lung cancer; these VOCs were detected using SPME-GC/MS from at least 83% of the exhaled breath of patients with lung cancer [14]. However, in our study, using GC/MS equipped with gas condenser unit, the only VOC selected as a candidate among the overlapping VOCs was toluene, and the list of identified VOCs in Table 2 did not include many overlapping VOCs as observed in previous reports. For acetone and isoprene, the average concentrations had been reported previously after analysis by SPME-GC/MS [39,42]. In our gas condenser-GC/MS study, almost all exhaled breath samples included these VOCs, as shown in Table 2. However, the average concentrations of acetone and isoprene were around 1/4–1/7 (gas condenser-GC/MS: 81.6 ppb; SPME-GC/MS: 358.6 ppb [39] and approximately 600 ppb from the histogram [42]) and 1/2 (gas condenser-GC/MS: 53.2 ppb; SPME-GC/MS: approximately 100 ppb from the histogram [39]), similar to those reposed in previous studies [39,42]. Based on these findings, for analysis of VOCs found at low concentrations in the exhaled breath, the gas condenser-GC/MS does not provide sufficient sensitivity relative to the SPME-GC/MS. However, the gas condenser-GC/MS could detect specific high concentrations of candidates, such as acids and acetoin, found in partial breath samples from patients with lung cancer. Thus, since a newly developed prototype adopted the same measurement principle involving GC and a gas condenser, this method may be useful for selection of specific high-concentration VOCs as target gases. Accordingly, we assumed that the results from the gas condenser-GC/MS were limited to development of the condenser, GC, and sensor-type prototype system.

### 3.3. Development of the Prototype System

As a criterion for the conceptualization of our prototype system, we set the conditions such that the measurement was completed within 10 min for screening use. The length of the GC column should be reduced for shorter screening times (10 min). In this case, the resolution of the retention time of each VOC was decreased compared with that of GC/MS, such that it was not possible to measure as many VOCs; thus, we installed two types of columns in order to detect more VOCs. However, it was still not possible to detect all 11 candidate VOCs on the prototype system, even if two types of columns were used. We therefore carried out correlation coefficient analysis to reduce the number of target VOCs and identify only the most important candidate VOCs. Table 3 shows the correlation coefficients of the four combinations with *r* > 0.6 from LC. Although the *r* value of the propanoic acid/acetoin pair in the LC-S group was greater than 0.6, the other pairs from HC and LC-S groups were less than 0.6. These pairs included nonanal, acetoin, acetic acid, and propanoic acid; therefore, we selected these four VOCs as the final VOCs for the prototype system.

**Figure 6:**
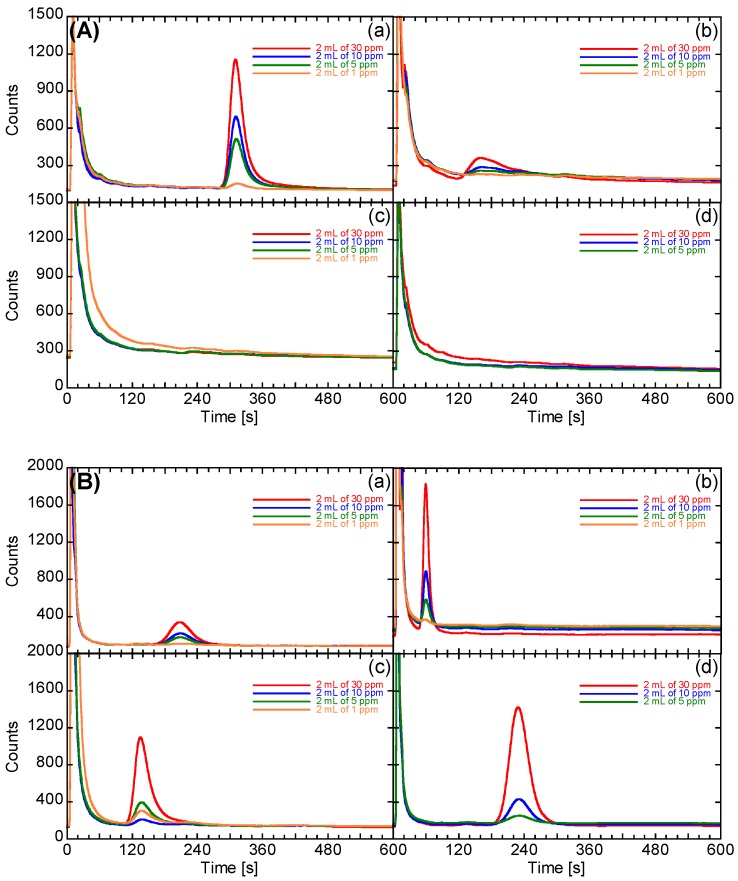
GC spectra from (**A**) column 1 and (**B**) column 2 of the prototype system used for analysis of several concentrations of: (**a**) nonanal; (**b**) acetoin; (**c**) acetic acid; and (**d**) propanoic acid. The condensed amounts of two milliliters of 1, 5, 10, and 30 ppm were almost the same as that of 200 mL (exhaled breath analysis) of 10, 50, 100, and 300 ppb VOCs, respectively.

Figure 6 shows the GC spectra of the prototype system used for measuring several concentrations of nonanal, acetoin, acetic acid, and propanoic acid. The first strong peaks resolved before 50 s were derived from moisture from sample gas. The combination between sensor 1 and GC column 1 for styrene monomer analysis could reveal fine nonanal peaks, for which the intensity was proportional to the concentration of nonanal. As described above, the original count value was empirically proportional to the concentration of VOCs. Therefore, the filling amount of the Tenax TA for condensing nonanal was sufficient, and condensed nonanal molecules reached sensor 1 without adsorption in the flowstream or GC column 1. Since GC column 1 was a styrene monomer column, we expected that this column would be suitable for separation of certain high-molecular-weight molecules, i.e., nonanal. In contrast, acetoin peaks were broad, and their intensities were not proportional to the concentration of acetoin. Acetic acid and propanoic acid were still present in the strong peaks at 50 s because of their low molecular weights. For the combination of sensor 2 and GC column 2 for analysis of short-chain fatty acids, the peaks of acetic acid and propanoic acid were fine, and the intensity was proportional to the concentration of acids. Moreover, the peaks of acetoin and nonanal were also fine. Because the peaks representing nonanal should overlap with those of propanoic acid, the concentrations of nonanal were analyzed based on the signal of sensor 1, and the concentrations of propanoic acid were analyzed based on the signal of sensor 2 after subtraction of the concentration of nonanal. Acetoin and acetic acid were also analyzed using sensor 2.

### 3.4. Evaluation of the Performance of the Prototype

**Figure 7:**
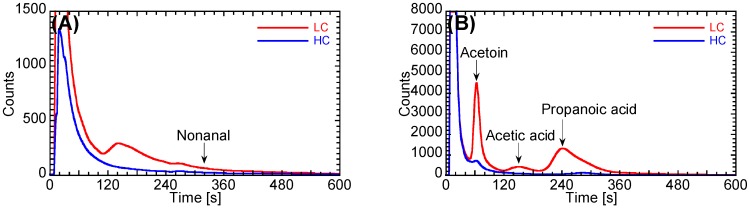
GC spectra from: (**A**) column 1; and (**B**) column 2 of the prototype system of exhaled breath samples from a patient with lung cancer (LC) and a healthy control (HC).

**Figure 8:**
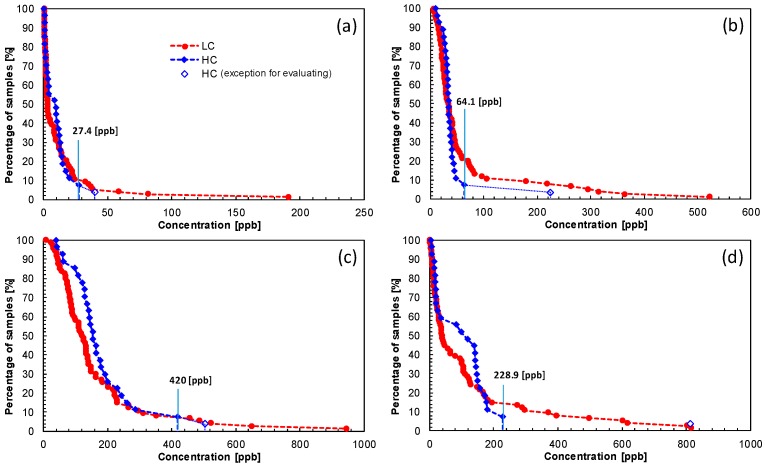
Percentages of samples having higher concentrations of: (**a**) nonanal; (**b**) acetoin; (**c**) acetic acid; and (**d**) propanoic acid on the prototype system. Open-rhombus plots from healthy controls were excluded from the evaluation of the *C*_cutoff_ because of abnormally high concentrations. LC: patients with lung cancer; HC: healthy controls.

Next, we evaluated the performance of the prototype system by comparison with the gas condenser-GC/MS. Figure 7 shows the GC spectra of the prototype system of the exhaled breath from one patient with lung cancer and one healthy control, and Figure 8 shows the percentages of samples having higher concentrations of four target gases. All concentrations for nonanal, acetoin, acetic acid, and propanoic acid from the prototype tended to be higher than those from the gas condenser-GC/MS analysis. According to the gas condenser-GC/MS results (Figure 5 and Table 2), exhaled breaths include many types of VOCs. The peaks from other VOCs may have overlapped with those from the four target VOCs, specifically nonanal. Based on the gas condenser-GC/MS analysis, the peak overlapped with nonanal may be associated with components having a similar retention time, such as tens ppb C_8_H_17_OH. Notably, the concentrations of the four target gases, as measured using the prototype system, may be higher than the actual concentrations because of the inclusion of other VOCs. We therefore evaluated the performance of the prototype system by comparison with the results of gas condenser-GC/MS using a percentage of LC samples that included VOCs at concentrations higher than the *C*_cutoff_ concentration, i.e., the maximum concentration from the HC group, in order to minimize the effects of other overlapped VOCs. Note that one exhaled breath sample from an HC included abnormally high concentrations of all target gases (open-rhombus plots in Figure 8). This particular HC did not exhibit any unique characteristics; however, we cannot predict whether this individual may exhibit disease features in the future. Importantly, the probability that this patient would have been identified as having lung cancer is very small if the results from this particular HC were included when defining the *C*_cutoff_. Therefore, the results from this HC were not included in the *C*_cutoff_ analysis in the present study. 

Table 4 shows the *C*_cutoff_ values of the four target VOCs and the number of samples that were classified as lung cancer on the prototype analysis. For comparison with the prototype analysis, Table 4 also includes the results of combinations using the four target VOCs from the gas condenser-GC/MS analysis.

Verification of the efficiency of the prototype system, excluding one HC sample due to erroneous measurements, revealed that the prototype analysis detected 10 of 74 samples (13.5% of LC samples). A sensitivity of 13.5% is not sufficient for screening. However, using the same cutoff analysis, gas condenser-GC/MS detected 14 of 107 samples (13.1% of LC samples), indicating that the prototype system exhibited a level of performance similar to that of the gas condenser-GC/MS system for breath analysis.

## 4. Conclusions

In our analysis using the gas condenser-GC/MS, the concentrations of 56 VOCs were identified in 136 breath samples exhaled by volunteers. By statistical analysis using *t*-tests, we selected 11 VOCs whose levels were significantly different between diseased and healthy lungs and between pre- and postsurgery states for development of the prototype system using the same measurement principle. Based on the results of the gas condenser-GC/MS analysis, we carried out correlation analyses to reduce the number of target VOCs for the prototype system and selected four target VOCs, i.e., nonanal, acetoin, acetic acid, and propanoic acid. The prototype system had a Tenax TA as the gas condenser unit, GC columns, and SnO_2_-based semiconductor gas sensors as detectors. The prototype utilized double GC columns and sensors for monitoring four VOCs within 10 min. When using the same evaluation conditions as those used in the cutoff method, the prototype system was found to have a performance level similar to that of the gas condenser-GC/MS analysis.

## Figures and Tables

**Table 1:** Number of volunteers, stage, and histology of lung cancer samples for gas condenser-GC/MS analysis.

	Number of Volunteers, n	Stage of Lung Cancer							Histology			
		IA	IB	IIA	IIB	IIIA	IIIB	IV	Ad	Sq	SCLC	Lg
LC	107	38	17	9	6	17	11	9	78	21	5	3
HC	29	–	–	–	–	–	–	–	–	–	–	–
LC-S	44	18	9	5	3	8	1	0	31	9	3	1

LC: patients with lung cancer; HC: healthy controls; LC-S: patients with lung cancer who had undergone resection; Ad: adenocarcinoma; Sq: squamous cell carcinoma; SCLC: small-cell lung cancer; Lg: large-cell carcinoma.

**Table 2:** VOCs identified in exhaled breaths and their average concentrations and standard deviations.

VOCs	LC (n = 107)			HC (n = 29)			LC-S (n = 44)		
	No. ^(1)^	Ave. (ppb)	Std. (ppb)	No.	Ave. (ppb)	Std. (ppb)	No.	Ave. (ppb)	Std. (ppb)
Hydrogen cyanide	57	1.7	2.3	20	1.6	1.0	19	0.8	0.9
Dimethylether	5	0.1	0.3	0	-	-	3	0.4	0.8
Methanol	107	54.5	71.1	29	46.6	29.6	44	27.5	16.6
Butane	26	0.9	2.1	14	1.3	1.3	19	1.4	2.3
C_n_F_2n+1_CO_2_H ^(2)^	31	0.9	1.2	6	0.5	0.8	1	0.1	0.2
Ethanol	107	80.3	37.3	29	102.1	88.5	44	75.6	47.2
Acetonitrile	8	3.7	17.9	7	6.2	29.5	1	2.3	14.2
Acetone	107	81.6	185.4	29	68.7	35.4	44	64.6	51.5
2-Propanol	97	3.2	6.5	24	3.5	6.2	42	3.1	1.2
Acrylonitrile	68	2.1	1.8	19	1.8	1.3	36	2.2	1.3
Isoprene	107	53.2	31.4	28	32.1	18.9	43	35.8	18.5
1,3-Pentadiene	106	17.9	10.7	28	10.6	5.1	43	13.9	10.3
Methyl acetate	1	0.2	1.1	0	-	-	2	0.2	0.3
C_5_H_6_^(2)^	0	-	-	1	0.1	0.3	0	-	-
1-Propanol	67	3.2	8.7	22	3.5	4.4	21	2.6	5.1
3,3-Dimethylpentan-2-one	2	0.1	0.4	0	-	-	0	-	-
Methyl ethyl ketone	1	0.0	0.2	0	-	-	1	0.1	0.4
2-Methylfuran	0	-	-	1	0.1	0.2	0	-	-
2-Propanoic acid	1	0.0	0.3	0	-	-	1	0.1	0.3
Ethyl acetate	36	0.8	1.0	8	0.7	0.9	22	2.5	9.9
Chloroform	47	1.0	1.4	11	1.2	1.7	20	0.9	0.6
Furan	1	0.2	2.4	0	-	-	0	-	-
Isopropyl alcohol	0	-	-	0	-	-	1	0.1	0.4
C_6_H_8_^(2)^	0	-	-	1	0.1	0.3	0	-	-
Acetic acid	11	1.4	5.6	0	-	-	2	0.3	1.3
Benzene	1	0.0	0.2	2	0.3	0.8	0	-	-
C_4_H_8_O_2_^(1)^	2	0.8	7.2	0	-	-	1	0.1	0.2
C_3_H_5_SCH_3_^(2)^	1	0.0	0.2	1	0.1	0.2	0	-	-
((CH_3_)_3_Si)_2_O	8	3.0	24.9	0	-	-	0	-	-
C_6_H_12_O_3_^(2)^	3	0.4	3.4	0	-	-	1	0.2	1.0
Acetoin	6	0.2	1.0	0	-	-	1	0.0	0.2
Ethyl propanoate	1	0.0	0.2	0	-	-	2	0.2	0.9
Heptane	3	0.1	0.3	1	0.1	0.2	1	0.1	0.2
C_3_H_7_SCH_3_^(2)^	9	0.3	0.7	6	0.5	1.0	4	0.1	0.3
Propanoic acid	12	1.5	3.7	2	0.2	0.6	4	0.9	2.5
C_3_H_5_SCH_3_^(2)^	15	0.4	0.9	4	0.5	1.2	1	0.2	0.5
C_5_H_11_OH ^(2)^	0	-	-	0	-	-	2	0.1	0.3
Methylcyclohexane	51	1.4	2.6	16	1.4	1.2	25	1.7	1.5
Toluene	98	3.5	2.8	29	6.3	5.4	40	4.8	3.2
C_8_H_16_^(2)^	15	0.5	0.5	4	0.6	0.6	0	-	-
C_8_H_16_^(2)^	91	2.5	1.7	28	2.7	1.6	30	1.5	1.0
C_8_H_16_^(2)^	69	1.6	1.1	18	1.7	1.1	18	0.9	0.7
Ethylcyclohexane	1	0.0	0.2	0	-	-	0	-	-
Xylene	6	0.4	0.5	3	0.6	0.6	0	-	-
Butyl ethyl ketone	1	0.2	1.8	0	-	-	0	-	-
C_7_H_15_OH ^(2)^	1	0.0	0.2	0	-	-	0	-	-
Nonane	1	0.0	0.2	0	-	-	0	-	-
α-Pinene	1	0.1	0.9	0	-	-	0	-	-
Dichlorobenzene	19	1.0	2.5	4	0.6	1.5	5	1.4	4.5
C_8_H_17_OH ^(2)^	105	29.5	17.5	29	36.0	14.4	43	17.2	13.8
Limonene	27	2.1	5.4	7	2.0	6.9	2	0.5	1.3
C_10_H_18_O ^(2)^	7	1.0	5.0	4	2.3	7.2	2	0.1	0.4
1-Methylstyrene	3	0.1	0.3	0	-	-	2	0.1	0.3
Nonanal	63	1.3	1.5	18	1.2	0.5	15	0.9	0.6
Undecane	33	2.2	5.0	7	3.2	6.2	24	5.1	7.7
Menthol	6	2.1	19.0	0	-	-	2	4.2	25.4

^(1)^ No.: number identified; Ave.: average concentration; Std.: standard deviation; ^(2)^ VOCs were identified using the mass database provided by Shimadzu. When a mass pattern of a peak was identified as several isomers, the VOC of the peak was expressed as a composition formula. LC: patients with lung cancer; HC: healthy controls; LC-S: patients with lung cancer who underwent surgery.

**Table 3:** Correlation coefficients of four pairs of VOCs.

Combinations of VOCs		Correlation Coefficient, *r*		
		LC	HC	LC-S
Acetic acid	Propanoic acid	0.60	-	0.19
Propanoic acid	Acetoin	0.73	-	0.90
Propanoic acid	Nonanal	0.64	0.15	−0.01
Acetoin	Nonanal	0.66	−0.10	−0.02

“–”: Either or both of the VOCs were not detected in all exhaled breath samples.

**Table 4:** Results of the simulation for detection of lung cancer from prototype analysis and gas condenser-GC/MS analysis using four target VOCs. The sample was classified as lung cancer when both VOCs were included with concentrations over the cutoff VOC concentrations.

Combinations of Target VOCs		From Prototype				From Gas Condenser-GC/MS			
		*C*_cutoff_ (ppb)		Number of Samples Classified		*C*_cutoff_ * (ppb)		Number of Samples Classified	
VOC A	VOC B	VOC A	VOC B	LC (n = 74)	HC (n = 27)	VOC A	VOC B	LC (n = 107)	HC (n = 29)
Nonanal	Acetoin	27.4	64.1	4	1	2.0	1.0	1	0
Nonanal	Acetic acid	27.4	420	2	1	2.0	1.0	2	0
Nonanal	Propanoic acid	27.4	229	4	1	2.0	2.5	3	0
Acetoin	Acetic acid	64.1	420	5	1	1.0	1.0	1	0
Acetoin	Propanoic acid	64.1	229	5	1	1.0	2.5	4	0
Acetic acid	Propanoic acid	420	229	2	1	1.0	2.5	8	0
Total **				10 (13.5%)	1 (3.7%)			14 (13.1%)	0 (0.0%)

* The minimum *C*_cutoff_ was set at 1.0 ppb because of the minimum limit of detection of the gas condenser-GC/MS. ** Total counts were not the sum of the number of samples because parts of samples overlapped with several classifications.
